# A Simple, Efficient Synthesis of 2-Aryl Benzimidazoles Using Silica Supported Periodic Acid Catalyst and Evaluation of Anticancer Activity

**DOI:** 10.1155/2013/453682

**Published:** 2013-04-24

**Authors:** Vyankat A. Sontakke, Sougata Ghosh, Pravin P. Lawande, Balu A. Chopade, Vaishali S. Shinde

**Affiliations:** ^1^Garware Research Centre, Department of Chemistry, University of Pune, Pune 411007, India; ^2^Institute of Bioinformatics and Biotechnology, University of Pune, Pune 411007, India

## Abstract

A new, efficient method for the synthesis of 2-aryl substituted benzimidazole by using silica supported periodic acid (H_5_IO_6_-SiO_2_) as a catalyst has been developed. The salient feature of the present method includes mild reaction condition, short reaction time, high yield and easy workup procedure. The synthesized benzimidazoles exhibited potent anticancer activity against MCF7 and HL60 cell lines.

## 1. Introduction

The benzimidazole nucleus is commonly present in a large number of natural products as well as pharmacologically active compounds [1]. It shows a wide spectrum of biological and pharmacological properties such as antifungal [2], antimicrobial [3], anthelmintic [4, 5], antiviral [6, 7], topoisomerase inhibition [8] and anticancer activities [9]. Some of their derivatives are marketed as antifungal drug (Carbendazim) [10], anthelmintic drug (Mebendazole and Thiabendazole) [11], antipsychotic drug (Pimozide) [12] and antiulcer agent (Omeprazole) [13]. Owing to their interesting pharmacological properties, great attention has been paid to the synthesis of benzimidazoles. Two main synthetic methods were well known in the literature. The most common method is direct condensation of 1,2-phenylenediamine and carboxylic acids [14, 15] or their derivatives [16], that require strong acidic conditions and sometimes need high temperature or the use of microwave [17]. The other synthetic route involves a two-step procedure that includes the cyclo-dehydrogenation of aniline Schiff's bases, which are often generated in situ from the condensation of 1,2-phenylenediamines and aldehydes [18], followed by oxidation with stoichiometric amount of oxidants, such as MnO_2_ [19], Oxone [20], NaHSO_3_ [21, 22], I_2_/KI/K_2_CO_3_/H_2_O [23] or catalytic use of CAN [24] and AIKIT-5 [25]. More recently, 2-alkyl substituted benzimidazoles are synthesized by using hexafluorophosphoric acid under microwave condition [26].

There is renewed interest in the silica supported catalyzed reactions [27]. These reactions have relatively shorter reaction time with high yield and cleaner chemistry. Moreover, the catalyst is easily separated from reaction mixture by simple filtration. There are very few reports involving solid supported catalyzed reaction for synthesis of benzimidazole derivatives. Jacob et al. [28] synthesized 1,2-disubstituted benzimidazoles by silica supported ZnCl_2_ catalyst that was found to be of poor yield. Patil et al. [29] developed a method for synthesis of 2-alkyl benzimidazoles using silica supported HBF_4_. Paul and Basu [30] described the synthesis of 1,2-disubstituted benzimidazoles by using silica gel soaked with Fe_2_(SO_4_)_3_·*x*H_2_O. Recently, Kumar et al. [31] reported silica supported HClO_4_ catalyzed synthesis of benzimidazoles.

**Scheme 1:**
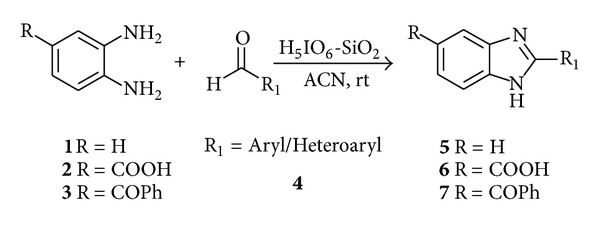
Synthesis of 2-aryl benzimidazole.

Periodic acid is an easily available hypervalent iodine reagent which is used in the oxidation of various functional groups [32, 33]. However, there are no reported efforts for the synthesis of benzimidazoles by using periodic acid. In this paper, we report an efficient and facile synthesis of 2-aryl benzimidazoles by using silica supported periodic acid (H_5_IO_6_-SiO_2_) as a catalyst (Scheme 1). Further, all synthesized derivatives were screened for anticancer activity against two cancer cell lines, namely, MCF7 and HL60. 

## 2. Result and Discussion

Herein, we used unprecedented silica supported periodic acid (H_5_IO_6_-SiO_2_) as catalyst for synthesis of 2-aryl benzimidazole derivatives. In our initial experiments, we choose 1,2-phenylenediamine (1 mmol) and *m-*nitrobenzaldehyde (1 mmol) as a model reaction for optimization of catalyst and reaction conditions. The results are summarized in Table 1. The use of 20 mol% of H_5_IO_6_ catalyst supported on silica resulted in 95% of desired product, **5 g** in 15 min at room temperature (Table 1, entry 1). Inferior yields were obtained on lowering the catalyst loading at room temperature (Table 1, entries 2 and 3). Even, on increasing temperature, the yield was not improved (Table 1, entries 6 to 8). In control experiments, the poor yields were found in the absence of H_5_IO_6_ (Table 1, entries 5 and 10) at room temperature and 60°C even after prolonged time (10 h). Further, the reactions were carried out with only H_5_IO_6_ without silica support at room temperature and 60°C (Table 1, entries 4 and 9) where the yield was not found to be more than 35%. These results confirmed that H_5_IO_6_ supported on silica significantly increased the efficacy of catalysts which may be attributed to the increase in available surface area. Thus, we found optimized conditions as the 1,2-phenylenediamine (1 mmol), aldehyde (1 mmol) and H_5_IO_6_ (0.20 mmol supported on silica) in acetonitrile (ACN) at room temperature.

Feasibility of the methodology was examined for a series of aryl/heteroaryl aldehydes bearing electron donating as well as electron withdrawing groups under the optimized reaction conditions and corresponding products were obtained in good to excellent yields (Table 2). Presence of electron withdrawing group in aldehyde system fastened the reaction (entries **5f**–**5h**) while opposite effect was observed for electron donating substituents and hindered aldehydes (entries **5b** and **5h**). We have not observed any remarkable change in the reaction time for the different substituted diamines. The reaction underwent smoothly even with aldehyde bearing two functional groups (entries **5c**,** 5h**,** 6c** & **7c**) and afforded corresponding products in good yields (entry **5h**). The reaction was carried out with substituted 1,2-phenylenediamine (entries **6a**–**6e** and **7a**–**7c**) and afforded 2,5-substituted benzimidazoles in moderate to good yields. Using these reaction conditions exclusively formation of 2-substituted benzimidazoles was observed. The products were characterized by their physical and spectral data. Thus, Table 2 illustrates generality and efficiency of this method for the synthesis of benzimidazoles.

**Scheme 2:**
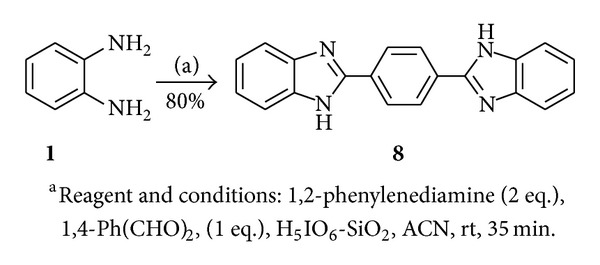
Synthesis of bisbenzimidazole.

We have also extended same methodology for the synthesis of bisbenzimidazole (**8**) derivative by using 1,2-phenylenediamine, aryl aldehyde (2 : 1) affording compound **8** in 80% yield (Scheme 2).

**Scheme 3:**
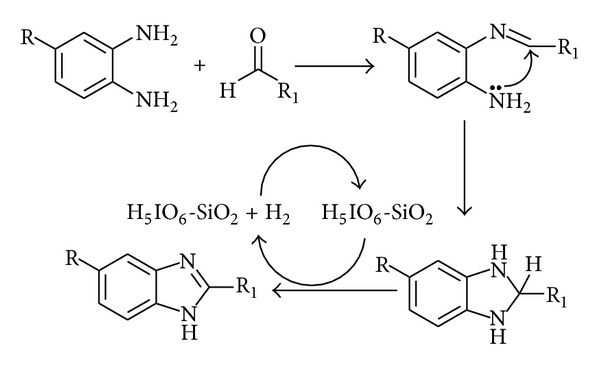
Plausible mechanism towards the formation of 2-aryl benzimidazole.

Although the exact mechanism is not clear, a proposed mechanism for the formation of benzimidazole is shown in Scheme 3. The actual oxidant is H_5_IO_6_-SiO_2_ and not SiO_2_ as confirmed by our controlled experiments (Table 1). The acidic site of catalyst is anticipated for oxidation [34].

## 3. Anticancer Activity

All the synthesized benzimidazoles were tested for their anticancer activity against two cell lines MCF7 (human breast adenocarcinoma) and HL60 (human promyelocytic leukemia) by MTT colorimetric assay using cisplatin as a standard anticancer drug. The results are expressed as IC_50_ in *μ*M and summarized in Table 2. Anticancer activity varies with substitution at 5-position of benzimidazole ring. The benzoyl substituted benzimidazole (**7a**–**7c**) showed the highest potency against two cell lines, while carboxyl substituted compounds (**6a** and **6b**) were moderately potent (with the exception of **6c**), as compared to unsubstituted benzimidazole (**5a**–**5c**). Dichloro derivatives (**5c **and** 6c**) exhibited more activity as compared to monochloro derivatives (**5d** and **6d**) against MCF7 and HL60. Substitution of nitro group (**5e**–**5g**) showed moderate and mostly similar effect for the given cell lines. Compound **5b** with phenolic −OH group (IC_50_ 27.63 *μ*M for MCF7 and 28.68 *μ*M for HL60) was comparable to **5h** (IC_50_ 29.67 *μ*M for MCF7 and 26.52 *μ*M for HL60) which has additional methoxy group at *p*-position. Replacing ring carbon of benzene ring with nitrogen atom as in **5i** (IC_50_ 30.42 *μ*M) showed better activity against MCF7 when compared with **5a** (IC_50_ 35.67 *μ*M), but same compound did not show substantial difference in activity against HL60. Bis-benzimidazole (**8**) was found to be more active for MCF7 (IC_50_ 17.45 *μ*M) than HL60 (IC_50_ 30.69 *μ*M). All the tested compounds are found to be more effective against both cell lines as compared to cisplatin.

## 4. Conclusion

We have developed a short and efficient method for the synthesis of 2-aryl benzimidazoles from 1,2-phenylenediamines and aryl aldehydes using H_5_IO_6_-SiO_2_ as catalyst. The mild reaction condition, low cost, easy workup procedure and good to excellent yields as well as the scope for using wide substrates make our methodology a valuable contribution to the existing processes for synthesis of benzimidazole derivatives. Among 18 derivatives, newly synthesized 5-substituted derivatives exhibited excellent activity against MCF7 and HL60 cell lines. The overall activities for all the derivatives tested were found in micromolar range. The current study provides better insight into the designing of more potent anticancer agents in the future.

## 5. Experimental Section

All reactions were performed in open atmosphere with unpurified reagents and distilled solvents. Periodic acid was purchased from Spectrochem. Acetonitrile and silica (230–400) were purchased from Sigma Aldrich. Thin-layer chromatography (TLC) was performed using 0.25 mm silica gel coated plates. Column chromatography was performed using the hexane: ethylacetate solvent and silica gel (60–120 meshes). ^1^H NMR (300 MHz) and ^13^C NMR (75 MHz) spectra were recorded on Varian Mercury instrument with DMSO-*d *_6_ or D_2_O as the solvents. Chemical shifts were reported in *δ* unit (ppm) with reference to TMS as an internal standard, and *J* values were given in Hertz. Melting points were determined on Thomas Hoover capillary melting point apparatus and are uncorrected. IR spectra were recorded on a Shimadzu FTIR 8400 spectrophotometer in KBr disc and expressed in cm^−1^. Elemental analysis was carried out with Thermo-Electron Corporation CHNS analyzer Flash-EA 1112.

### 5.1. Cell Culture

Two cancer cell lines, MCF7 (human breast adenocarcinoma) and HL60 (human promyelocytic leukemia), were obtained from National Center for Cell Sciences, India. MCF7 was cultured in DMEM medium [35] while HL60 cells were cultured in a humidified atmosphere (37°C, 5% CO_2_) in RPMI1640 medium supplemented with 10% fetal bovine serum.

### 5.2. MTT Assay

Test compounds were evaluated for anticancer activity against two cancer cell lines using cisplatin as standard anticancer drug. The compounds were evaluated* in vitro* at a concentration range of 10 *μ*M to 100 *μ*M. The MTT colorimetric assay was used to determine growth inhibition. 100 *μ*L of cell suspension (5 × 10^6^ cells) were plated in 96-well plates and allowed to attach for 24 h. The compounds were dissolved in 0.5% DMSO. Cells were exposed in triplicate wells to these derivatives at various concentrations for 48 h. After 48 h, 20 *μ*L MTT (3-(4,5-dimethylthiazol-2-yl)-2,5-diphenyltetrazolium bromide) solution (5 mg/mL) was added to each well. After 1 h of incubation, the solution was centrifuged for 5 min under 4000 rpm, and the medium was discarded carefully. The formazan precipitate was dissolved in DMSO (200 *μ*L), then shaken by oscillator. The absorbance at 570 nm was determined on a microplate reader (Bio-Rad Model 3350, Japan). The absorbance values were used to calculate % inhibition at various concentrations and IC_50_ values.

### 5.3. Procedure for Synthesis of Silica Supported H_5_IO_6_ Catalyst (H_5_IO_6_-SiO_2_)

H_5_IO_6_ (2.50 g, 10.96 mmol) was dissolved in 15 mL of hot water (70°C) in a 50 mL round-bottomed flask. To the hot solution was added silica gel (230–400 meshes, 10 g) with vigorous stirring. The resultant H_5_IO_6_ (resultant mixture contains 20 wt% of H_5_IO_6_) supported with silica gel was dried in oven at 100°C for 12 h to obtain a white free flow powder. The reagent can be stored for 4 months with negligible loss of activity.

### 5.4. General Procedure for Synthesis of Benzimidazoles (**5a–5i**, **6a–6e** and **7a–7c**)

A typical procedure is as follows. A mixture of 1,2-phenylenediamine (108 mg, 1.0 mmol), *m*-nitrobenzaldehyde (151 mg, 1.0 mmol) in acetonitrile (3.0 mL) was taken, and H_5_IO_6_ (45 mg, 20 mmol% supported on silica 210 mg) was added at room temperature. The reaction was stirred at room temperature for 15 minutes. After completion of the reaction (monitored by TLC), filter the reaction mixture over celite. The filtrate was evaporated under vacuum and subsequently dried to afford crude product which was purified by column chromatography using hexane/ethylacetate as eluent to afford pure benzimidazole **5g** (227 mg, 95%). 

### 5.5. Procedure for Synthesis of Bisbenzimidazole (**8**)

A mixture of 1,2-phenylenediamine (216 mg, 2.0 mmol), *p*-phthalaldehyde (134 mg, 1.0 mmol) in acetonitrile (3.0 mL) was taken, and H_5_IO_6_ (90 mg, 40 mol% supported on silica 420 mg) was added at room temperature. The reaction was stirred at room temperature for 35 minutes. After completion of the reaction (monitored by TLC), the reaction mixture was filtered over celite. The filtrate was evaporated under vacuum and subsequently dried to afford crude product which was purified by column chromatography using hexane/ethylacetate as eluent to afford pure benzimidazole **8** (250 mg, 80%). The spectral data are in full agreement with data reported in the literature. Spectral data of compounds are given below.

#### 5.5.1. 2-Phenyl-1H-benzoimidazole (**5a**)

White solid; mp 291–293°C; (lit. [21, 22] mp 290-291°C); IR (cm^−1^, KBr): 3044, 1622, 1587, 1537, 1458, 1439, 1407, 1312, 1274; ^1^H NMR (300 MHz, DMSO-*d *_6_): *δ* 12.91 (brs, 1H), 8.15 (d, *J* = 7.0 Hz, 2H), 7.55–7.47 (m, 5H), 7.19 (brs, 2H); ^13^C  NMR (75 MHz, DMSO-*d *_6_): *δ* 151.2, 143.7, 134.9, 130.1, 129.8, 128.9, 126.4, 122.4, 121.6, 118.8, 111.3; (Found: C, 80.39; H, 5.18; N, 14.38. Cal for C_13_H_10_N_2_: C, 80.42; H, 5.19; N, 14.42%).

#### 5.5.2. 2-(1H-Benzo[d]imidazol-2-yl) Phenol (**5b**)

White solid; mp 235–237°C; (lit. [21, 22] mp 236-237°C); IR (cm^−1^, KBr): 3327, 3057, 2332, 1635, 1280, 1037, 840, 729; ^1^H NMR (300 MHz, DMSO-*d *_6_): *δ* 13.21 (brs, 2H), 8.07 (d, *J *= 7.7 Hz, 1H), 7.67 (brs, 2H), 7.28–7.40 (m, 3H), 6.99–7.06 (m, 2H); ^13^C NMR (75 MHz, DMSO-*d *_6_): *δ* 158.0, 151.7, 131.6, 126.2, 122.7, 119.0, 117.1, 112.5; (Found: C, 74.25; H, 4.78; N, 13.31. Cal for C_13_H_10_N_2_O: C, 74.27; H, 4.79; N, 13.33%).

#### 5.5.3. 2-(2,6-Dichlorophenyl)-1H-benzimidazole (**5c**)

White solid; mp 274–276°C; (lit. [36] mp 275-276°C); IR (cm^−1^, KBr): 3368, 3297, 1558, 1431, 1369, 1265, 1132, 735; ^1^H NMR (300 MHz, DMSO-*d *_6_): *δ* 12.90 (brs, 1H), 7.53–7.71 (m, 5H), 7.20–7.29 (m, 2H); ^13^C NMR (75 MHz, DMSO-*d *_6_): *δ* 146.7, 143.1, 135.0, 134.0, 132.3, 130.5, 128.3, 122.8, 121.6, 119.2, 111.6; (Found: C, 59.33; H, 3.05; N, 10.62. Cal for C_13_H_8_Cl_2_N_2_: C, 59.34; H, 3.06; N, 10.65%).

#### 5.5.4. 2-(4-Chlorophenyl)-1H-benzimidazole (**5d**)

White solid; mp 288–291°C; (lit. [25] 287–289°C); IR (cm^−1^, KBr) 3433, 3055, 1427, 1273, 1091, 829, 744; ^1^H NMR (300 MHz, DMSO-*d *_6_,): *δ* 13.00 (brs, 1H), 8.23 (d, *J* = 8.2 Hz, 2H), 7.63 (d, *J *= 8.5 Hz, 2H), 7.54 (d, *J *= 5.9 Hz, 2H), 7.21 (brs, 2H); ^ 13^C NMR (75 MHz, DMSO-*d *_6_): *δ* 150.2, 143.7, 134.5, 129.1, 129.0, 128.1, 122.4, 118.9, 111.5; (Found: C, 68.27; H, 3.95; N, 12.21. Cal for C_13_H_9_ClN_2_: C, 68.28; H, 3.97; N, 12.25%).

#### 5.5.5. 2-(4-Nitrophenyl)-1H-benzo[d]imidazole (**5e**)

Yellow solid; mp 300–302°C; (lit. [21, 22] mp 299–301°C); IR (cm^−1^, KBr): 3335, 2912, 1602, 1514, 1435, 1340, 1103, 856, 746; ^1^H NMR (300 MHz, DMSO-*d *_6_): *δ* 13.39 (brs, 1H), 8.38–8.27 (m, 4H), 7.61 (s, 2H), 7.22–7.20 (m, 2H); ^13^C NMR (75 MHz, DMSO-*d *_6_): *δ* 148.8, 147.5, 142.7, 135.9, 127.2, 124.1, 124.0, 122.8; (Found: C, 65.25; H, 3.75; N, 17.52. Cal for C_13_H_9_N_3_O_2_: C, 65.27; H, 3.79; N, 17.56%).

#### 5.5.6. 2-(2-Nitrophenyl)-1H-benzo[d]imidazole (**5f**)

Yellow solid; mp 209–211°C; (lit. [25] 208–210°C); IR (cm^−1^, KBr): 3410, 3078, 2686, 1525, 1348, 1078, 972, 746; ^1^H NMR (300 MHz, DMSO-*d *_6_): *δ* 13.07 (brs, 1H), 7.96–8.04 (m, 2H), 7.83–7.89 (m, 1H), 7.72–7.78 (m, 1H), 7.60–7.63 (m, 2H), 7.23–7.26 (m, 2H); ^13^C NMR (75 MHz, DMSO-*d *_6_): *δ* 148.9, 147.2, 132.5, 131.4, 130.8, 130.8, 128.6, 124.2, 122.4, 119.1, 111.5; (Found: C, 65.26; H, 3.74; N, 17.53. Cal for C_13_H_9_N_3_O_2_: C, 65.27; H, 3.79; N, 17.56%).

#### 5.5.7. 2-(3-Nitrophenyl)-1H-benzimidazole (**5g**)

Yellow solid; mp 205–207°C; (lit. [21, 22] mp 205-206°C); IR (cm^−1^, KBr): 3371, 3088, 1587, 1518, 1473, 1429, 1346, 1274; ^1^H NMR (300 MHz, DMSO-*d *_6_) *δ* 13.29 (s, 1H), 9.01 (s, 1H), 8.60 (d, *J* = 7.6 Hz, 1H), 8.32 (d, *J *= 7.9 Hz, 1H), 7.84 (t, *J *= 7.9 Hz, 1H), 7.72 (d, *J *= 7.3 Hz, 1H), 7.58 (d, *J* = 7.4 Hz, 1H), 7.25 (t, *J* = 6.7 Hz, 2H); ^13^C NMR (75 MHz, DMSO-*d *_6_): *δ* 148.9, 148.2, 143.5, 134.9, 132.3, 131.6, 130.5, 124.0, 123.1, 122.0, 120.7, 119.1, 111.5; (Found: C, 66.26; H, 3.75, N; 17.55. Cal for C_13_H_9_N_3_O_2_: C, 65.27; H, 3.79; N, 17.56%).

#### 5.5.8. 5-(1H-Benzo[d]imidazol-2-yl)-2-methoxyphenol (**5h**)

Yellow solid; mp: 218–220°C; IR (cm^−1^, KBr): 3273, 2926, 1500, 1450, 1265, 1033, 910, 736; ^1^H NMR (300 MHz, DMSO-*d *_6_): *δ* 12.65 (s, 1H), 9.31 (s, 1H), 7.52–7.63 (m, 4H), 7.13–7.19 (m, 2H), 7.05 (d, *J *= 8.2 Hz, 1H), 3.85 (s, 3H); ^13^C NMR (75 MHz, DMSO-*d *_6_): *δ* 151.5, 149.3, 146.6, 139.9, 122.8, 121.6, 117.9, 114.4, 113.7, 112.0, 55.6; (Found: C, 69.91; H, 4.99; N, 11.65. Cal for C_14_H_12_N_2_O_2_: C, 69.99; H, 5.03; N, 11.66%).

#### 5.5.9. 2-(Pyridine-2-yl)-1H-benzo[d)imidazole (**5i**)

Yellow solid; mp 218–220°C; IR (cm^−1^, KBr): 3057, 2667, 1595, 1444, 1315, 1280, 1122, 850, 744, 615; ^1^H NMR (300 MHz, DMSO-*d *_6_): *δ* 13.11 (brs, 1H), 8.72 (d, *J *= 4.8 Hz, 1H), 8.33 (d, *J *= 8.1 Hz, 1H), 8.02 (t, *J *= 7.7 Hz, 1H), 7.49–7.69 (m, 3H), 7.21–7.24 (m, 2H); ^13^C NMR (75 MHz, DMSO-*d *_6_): *δ* 151.2, 150.1, 148.3, 138.3, 125.6, 123.6, 122.0, 116.2; (Found: C, 78.80; H, 4.60; N, 21.51. Cal for C_12_H_9_N_3_: C, 78.83; H, 4.65; N, 21.52%).

#### 5.5.10. 2-Phenyl-1H-benzo[d]imidazole-5-carboxylic Acid (**6a**)

White solid; mp 301–303°C; IR (cm^−1^, KBr): 3405, 1680, 1621, 1450, 981, 770; ^1^H NMR (300 MHz, DMSO-*d *_6_): *δ* 13.18 (brs, 1H), 12.79 (brs, 1H), 8.19 (m, 3H), 7. 84 (d, *J* = 8.5 Hz, 1H), 7.63 (d, *J* = 15.2 Hz, 1H), 7.58–7.48 (m, 3H); ^13^C NMR (75 MHz, DMSO-*d *_6_): *δ* 193.2, 167.8, 153.5, 134.5, 130.4, 129.5, 129.2, 129.0, 128.5, 126.7, 124.6, 123.6, 117.1, 114.6; (Found: C, 70.55; H, 4.24; N, 11.72. Cal for C_14_H_10_N_2_O_2_: C, 70.58; H, 4.23; N, 11.76%).

#### 5.5.11. 2-(2-Hydroxyphenyl)-1H-benzo[d]imidazole-5-carboxylic Acid (**6b**)

White solid; mp 301–303°C; IR (cm^−1^, KBr): 3319, 3059, 1681, 1633, 1491, 1261, 1130, 748; ^1^H NMR (300 MHz, DMSO-*d *_6_): *δ* 13.43 (brs, 1H), 12.84 (brs, 2H), 8.27 (brs, 1H), 8.10 (d, *J* = 7.2 Hz, 1H), 7.90 (brs, 1H), 7.72 (brs, 1H), 7.43 (t, *J* = 7.4 Hz, 1H), 7.07 (d, *J* = 8.6 Hz, 2H); ^13^C NMR (75 MHz, DMSO-*d *_6_): *δ* 168.3, 158.0, 153.9, 132.9, 132.0, 129.1, 127.3, 125.5, 124.6, 120.1, 117.6, 112.8; (Found: C, 66.16; H, 3.93; N, 10.09. Cal for C_14_H_10_N_2_O_3_: C, 66.14; H, 3.96; N, 11.02%).

#### 5.5.12. 2-(2,6-Dichlorophenyl)-1H-benzo[d]imidazole-5-carboxylic Acid (**6c**)

White solid; mp 304–306°C; IR (cm^−1^, KBr): 3171, 1915, 1668, 1622, 1433, 1315, 779; ^1^H NMR (300 MHz, DMSO-*d *_6_): 13.12 (brs, 1H), 12.64 (brs, 1H), 8.28–8.17 (m, 1H), 7.88 (d, *J* = 7.6 Hz, 1H), 7.72–7.57 (m, 4H); ^13^C NMR (75 MHz, DMSO-*d *_6_): *δ* 167.7, 167.3, 134.9, 134.6, 132.6, 132.3, 130.0, 129.7, 128.4, 128.1; (Found: C, 54.71; H, 2.59; N, 9.14. Cal for C_14_H_8_Cl_2_N_2_O_2_: C, 54.75; H, 2.63; N, 9.12%).

#### 5.5.13. 2-(4-Chlorophenyl)-1H-benzo[d]imidazole-5-carboxylic Acid (**6d**)

White solid; mp 194–196°C; IR (cm^−1^, KBr): 3090, 2821, 1907, 1676, 1624, 1425, 1319, 1026, 947, 835, 731; ^1^H NMR (300 MHz, DMSO-*d *_6_): *δ* 13.30 (brs, 1H), 12.77 (brs, 1H), 8.19 (m, 3H), 7.84 (brs, 1H), 7.65 (m, 3H); ^13^C NMR (75 MHz, DMSO-*d *_6_): *δ* 167.8, 152.4, 135.0, 129.1, 128.4, 128.4, 124.7, 123.8, 122.4, 119.9; (Found: C, 61.65; H, 3.31; N, 10.25. Cal for C_14_H_9_ClN_2_O_2_: C, 61.66; H, 3.33; N, 10.27%).

#### 5.5.14. 2-(4-Nitrophenyl)-1H-benzo[d]imidazole-5-carboxylic Acid (**6e**)

Yellow solid; mp 272–274°C; IR (cm^−1^, KBr): 3338, 2949, 1699, 1604, 1514, 1348, 1213, 853, 774; ^1^H NMR (300 MHz, DMSO-*d *_6_,): *δ* 13.38 (s, 2H), 8.39 (s, 4H), 8.20 (s, 1H), 7.86 (d, *J* = 8.1 Hz, 1H), 7.69 (d, *J* = 8.1 Hz, 1H); ^13^C NMR (75 MHz, DMSO-*d *_6_): *δ* 168.0, 151.5, 148.4, 142.3, 139.5, 135.4, 131.8, 128.9, 128.0, 125.6, 124.5, 117.9, 115.3; (Found: C, 59.32; H, 3.15; N, 14.81. Cal for C_14_H_9_N_3_O_4_: C, 59.37; H, 3.20; N, 14.84%).

#### 5.5.15. Phenyl(2-phenyl-1H-benzo[d]imidazol-5-yl)methanone (**7a**)

Yellow solid; mp 221-222°C; IR (cm^−1^, KBr): 3375, 3061, 1645, 1572, 1321, 902, 707; ^1^H NMR (300 MHz, DMSO-*d *_6_): *δ* 13.30 (s, 1H), 8.20 (d, *J* = 6.2 Hz, 2H), 7.94 (brs, 1H), 7.76–7.54 (m, 10H); ^13^C NMR (75 MHz, DMSO-*d *_6_): *δ* 195.5, 138.0, 132.0, 130.9, 130.4, 129.4, 129.0, 128.3, 126.6, 124.2; (Found: C, 80.43; H, 4.65; N, 9.31. Cal for C_20_H_14_N_2_O: C, 80.52; H, 4.73; N, 9.39%).

#### 5.5.16. 2-(2-Hydroxyphenyl)-1H-benzo[d]imidazol-5-yl)(phenyl)methanone (**7b**)

Light yellow solid; mp 227–229°C; IR (cm^−1^, KBr): 3298, 3057, 1919, 1726, 1614, 1450, 1294, 981, 786; ^1^H NMR (300 MHz, DMSO-*d *_6_): *δ* 12.91 (brs, 2H), 8.10 (d, *J *= 8.1 Hz, 1H), 8.08 (brs, 1H), 7.81–7.75 (m, 4H), 7.69 (m, 1H), 7.58 (m, 2H), 7.42 (m, 1H), 7.08–7.02 (m, 2H); ^13^C NMR (75 MHz, DMSO-*d *_6_): *δ* 196.5, 158.0, 154.3, 138.3, 133.0, 132.8, 131.9, 130.0, 129.0, 127.4, 135.3, 120.2, 117.7, 113.0; (Found: C, 76.41; H, 4.42; N, 8.88. Cal for C_20_H_14_N_2_O_2_: C, 76.42; H, 4.49; N, 8.91%).

#### 5.5.17. 2-(2,6-Dichlorophenyl)-1H-benzo[d]imidazol-5-yl)(phenyl)methanone (**7c**)

White solid; mp 164–166°C; IR (cm^−1^, KBr): 3059, 1734, 1651, 1431, 1317, 970,788; ^1^H NMR (300 MHz, DMSO-*d *_6_): *δ* 13.29 (brs, 1H), 8.05 (brs, 1H), 7.74 (m, 3H), 7.70–7.64 (m, 5H), 7.58 (t, *J *= 7.6 Hz, 2H); ^13^C NMR (75 MHz, DMSO-*d *_6_): *δ* 195.7, 148.9, 137.8, 134.8, 132.6, 132.0, 129.9, 129.4, 128.3, 120.0; (Found: C, 65.38; H, 3.25; N, 7.70. Cal for C_20_H_12_Cl_2_N_2_O: C, 65.41; H, 3.29; N, 7.63%).

#### 5.5.18. 2-(4-(1H-Benzo[d]imidazol-2-yl)phenyl)-1H-benzo[d]imidazole (**8**)

White solid; mp 245–247°C; IR (cm^−1^, KBr): 3061, 1626, 1440, 1317, 1118, 966, 846, 740; ^1^H NMR (300 MHz, DMSO-*d *_6_): *δ* 13.01 (brs, 2H), 8.33 (s, 4H), 7.61 (s, 4H), 7.23-7.20 (m, 4H); ^13^C NMR (75 MHz, DMSO-*d *_6_): *δ* 150.3, 138.9, 130.7, 127.0, 122.6, 115.1; (Found: C, 77.31; H, 4.54; N, 17.98. Cal for C_20_H_14_N_4_: C, 77.40; H, 4.55; N, 18.05%).

## Figures and Tables

**Table 1:** Reaction of **1** and **4g** under various conditions^a^.

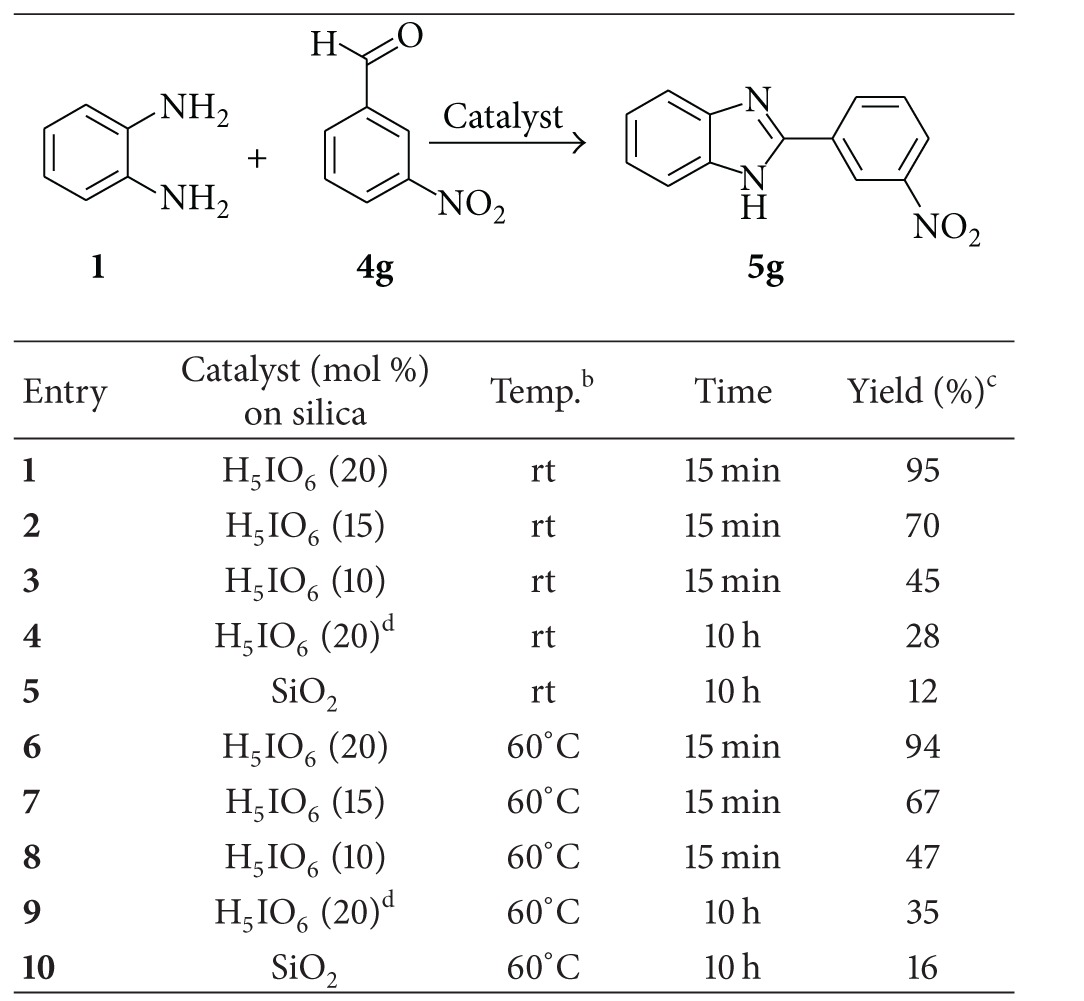

^a^1,2-phenylenediamine 1 mmol, *m*-nitrobenzaldehyde 1 mmol catalyst (0.20 mmol supported on silica) in ACN.

^b^Room temperature was 30–35°C.

^c^Yields are measured after purification.

^d^H_5_IO_6_ which is not supported on silica.

**Table 2:** Synthesis and anticancer activity of 2-aryl benzimidazoles against MCF7 and HL60 cell lines.

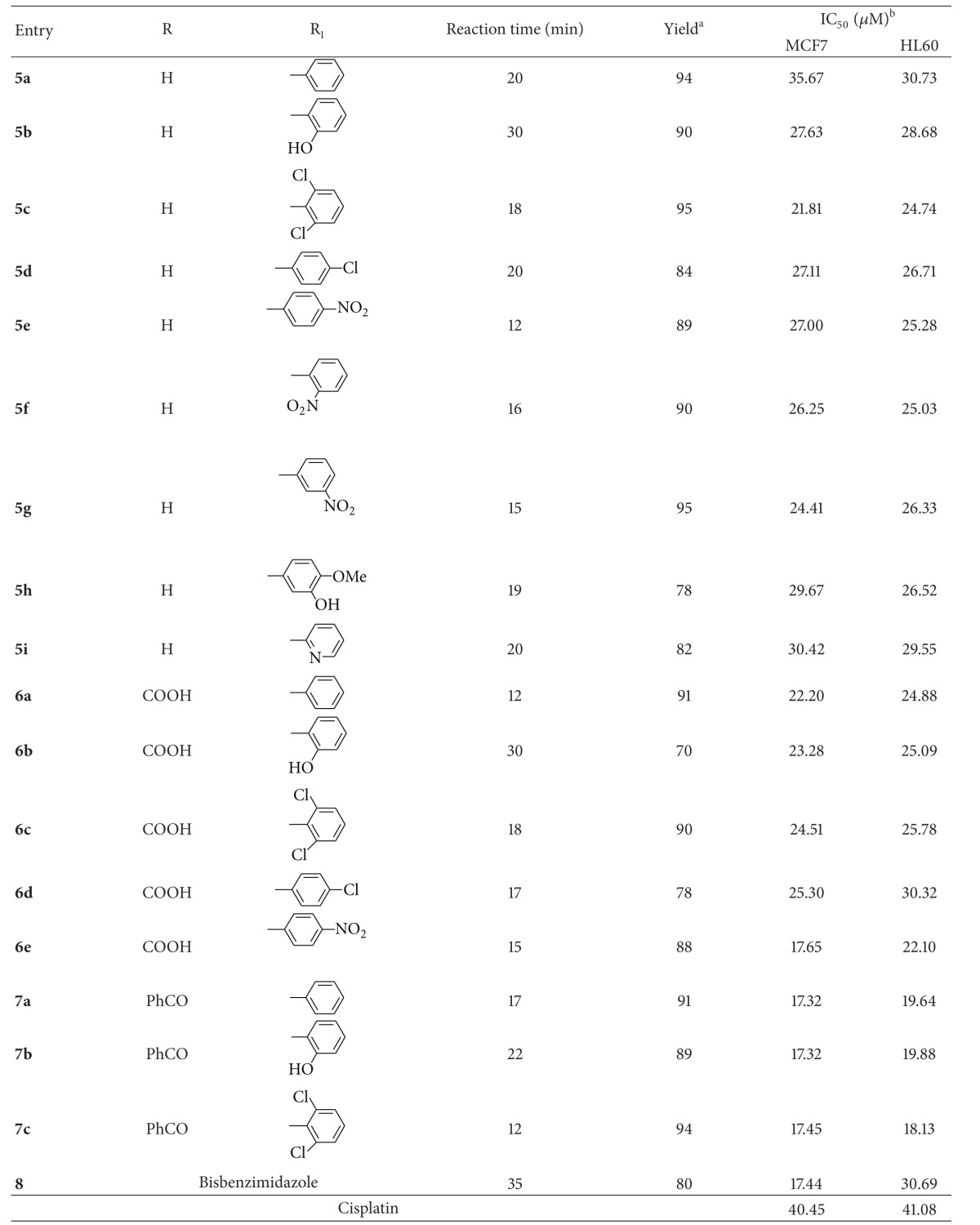

^a^1,2-phenylenediamine 1 mmol, *m*-nitrobenzaldehyde 1 mmol catalyst (0.20 mmol supported on silica) in ACN. Isolated yields after purification.

^b^IC_50_ 50% inhibition concentration in *μ*M.
